# Dr. Latta’s Pamphlet on Homœopathy

**Published:** 1850-08

**Authors:** 


### DR. LATTA'S PAMPHLET ON HOMOEOPATHY.

A notice of Dr. Latta's excellent pamphlet on the allopathic doses of falsehood, given by the infinitesimal gentlemen of Cincinnati, has been crowded out of our two last numbers. The pamphlet is an able and convincing document, and will well repay several perusals. It is very clear that Dr. Latta's faith in Homœopathic truth is of a Homœopathic cast. It seems to have been triturated until it has reached an attenuation that would require one of Chevalier's microscopes to find. The Doctor lashes the heresy with an unsparing hand, and disrobes it to perfect nudity.

We thank Dr. Latta for his able and conclusive expose of the miserable claims set up by Homœopathy in the treatment of cholera, or of any other diseases. We speak now of the principles of Hanneman, not of the *practice* of all those who call themselves his disciples. We know gentlemen of that ilk for whom we entertain a sincere respect. They are scholars, and men of mind, and they know enough of the fallacies of Homœopathy to know when and how far to trust to it. B.

